# Anti-inflammatory and antioxidant efficacy of lavender oil in experimentally induced thrombosis

**DOI:** 10.1186/s12959-023-00516-0

**Published:** 2023-08-09

**Authors:** Valeriu Mihai But, Adriana Elena Bulboacă, Vasile Rus, Tamás Ilyés, Mădălina Luciana Gherman, Sorana D. Bolboacă

**Affiliations:** 1 Department of Medical Informatics and Biostatistics, “Iuliu Haţieganu” University of Medicine and Pharmacy, Louis Pasteur Street, No. 6, Cluj-Napoca, 400349 Romania; 2 Department of Pathophysiology, “Iuliu Haţieganu” University of Medicine and Pharmacy, Victor Babeş Street, No. 2-4, Cluj-Napoca, 400012 Romania; 3 Department of Cell Biology, Histology and Embryology, University of Agricultural Sciences and Veterinary Medicine, Cluj-Napoca, 400374 Romania; 4 Department of Medical Biochemistry, “Iuliu Haţieganu” University of Medicine and Pharmacy, Louis Pasteur Street, No. 6, Cluj-Napoca, 400349 Romania; 5 Experimental Center, “Iuliu Haţieganu” University of Medicine and Pharmacy, Cluj-Napoca, 400012 Romania

**Keywords:** Lavender oil, Thrombosis, Anti-inflammatory agents, Oxidative stress

## Abstract

**Background:**

Lavender oil (LO) possesses anti-inflammatory, antioxidant, antifungal, antibacterial, sedative, cardio-protective, and antinociceptive properties. Thrombosis and inflammation are interplayed processes that interact and influence one another. Our research compared three routes of administration to assess the efficacy of pretreatment with LO on carrageenan-induced thrombosis in rat tail.

**Materials and methods:**

Wistar-Bratislava white rats were randomly divided into five groups of ten rats each and pretreated 3 consecutive days prior the inducement of thrombosis to with one dose of LO (150 mg/kg body weight (b.w.)): *per os* by gavage (TLOPO group), intraperitoneal (TIPLO group) and subcutaneous (TSCLO group). We also have a control (C, received saline solution 0.9% and DMSO (vehicle) 1 ml intraperitoneal (i.p.)) group and a group with thrombosis (T group, received saline solution 0.9% plus vehicle 1 ml i.p.). Histopathological examinations were conducted together with measurements of the circulating levels of three oxidative stress markers, antioxidant effect (TAC and THIOL), and three proinflammatory cytokines (TNF- α, RANTES, and MCP-1).

**Results:**

When administered intraperitoneally, lavender oil has the best efficacy on circulating levels of oxidative stress parameters (MDA, NOx, TOS), one oxidative stress marker (THIOL), and all studied proinflammatory cytokines (p-values < 0.02). Moreover, TIPLO displayed the closest values for bleeding and clotting time to the C group, as well as the lowest length of the thrombus than the T, TPOLO, and TSCLO groups (p-values < 0.001). The TIPLO group has histological appearance comparable to the C group, with the exception of the presence of oedema.

**Conclusions:**

Lavender oil pretreatment with intraperitoneal administration as three days, one-dose per day, showed anti-inflammatory and antioxidant efficacy in experimentally induced thrombosis.

## Introduction

Lavender (*Lavandula angustifolia*) belongs to the family Labiatae (*Lamiacae*) and is one of the world’s most widely used aromatic plants. Lavender has been used in traditional medicine for centuries as a herbal remedy [1]. Extracts and essential oils of *Lavandula angustifolia* were traditionally used to treat conditions such as epilepsy and migraine and to reduce spasms in colic pain [2]. The Lavender oil proved to contain over 100 chemical constituents, the main components being linalool, linalyl acetate, α-pinene, limonene, 1,8-cineole, cis- and trans-ocimene, 3-octanone, caryophyllene, camphor, terpinen-4-ol and lavendulyl acetate, and flavonoids [3]. It has been demonstrated that its major components, linalool and linalyl acetate, are linked to the anti-inflammatory activity of lavender essential oil [4]. Furthermore, various studies have shown that linalool and linalyl acetate have significant antioxidant, antimicrobial, and sedative effects. It has been observed that linalool reduces inflammation, enhances immune system function, and protects against oxidative stress [5]. Linalyl acetate was also discovered to have anti-inflammatory and antibacterial effects [6]. The effects of lavender oil extracted from *Lavandula hybrida*, which also contains linalool and linalyl acetate as its primary components, on platelet aggregation were studied in vitro. The lavender oil completely prevented the aggregation of platelets [7].

Hemostasis balance and interplay between procoagulant and anticoagulant molecules have an important role in thrombus formation. Thrombus formation starts with platelet activation and is followed by platelet adhesion to the affected subendothelium. They secrete growth factors, aggregation activators, and procoagulant substances. Platelets promote vascular repair but also amplify the coagulation response and clot formation. Previous studies have shown that activation of blood platelets leads to the release of mediators with pleiotropic functions in inflammation. Thus, inflammation can cause arterial and venous thrombosis, provoking advanced clinical disease often. Inflammation is also the pathophysiological process that causes diseases that, in their progression, are often aggravated by developing thrombosis [8]. The interrelationship between thrombosis and inflammation is of interest in current studies, and these processes become more evidently intricate with aging. Inflammation promotes aggregation and coagulation, while activated platelets and coagulation factors or peptides promote inflammation by several mechanisms [9]. The existing evidence in the literature points to the role played by platelets and possibly clotting factors in regulating innate immunity, and the latest findings introduce new concepts such as immunothrombosis and thromboinflammation [10]. Several distinct components mediates platelet adhesion to the subendothelium, glycoproteins such as von Willebrand factor (VWF), fibrinogen, and collagen, as well as GPIIb/IIIa and GPVI receptors. The GPIIb/IIIa receptor binds fibrinogen, which stimulates platelet activation, while the GPVI receptor binds collagen and stimulates the release of pro-inflammatory mediators [11]. The onset of venous thrombosis is caused by decreased blood flow velocity, which causes abnormal activation of immunothrombosis, generating the coagulation cascade. In veins, along with vascular endothelium lesions, reduced blood flow velocity activates mast cells in the releasing histamine and signaling endothelial cells to mobilize adhesion molecules P-selectin and von Willebrand factor to their surface. Platelets and innate immune cells are attracted to the endothelium surface, which is supported by the binding of all-thiol high mobility group protein B1 (HMGB1) produced from platelets to the receptor for advanced glycation end products and Toll-like receptor 2 on monocytes and CXC chemokine receptor 2 on neutrophils. Platelet-derived oxidized HMGB1 activates monocytes to generate pro-inflammatory mediators, boosting innate immune cell activation [9].

Platelets may detect infectious pathogens s by way of their Toll-like receptors. Infections can cause a pro-inflammatory platelet response, releasing pro-inflammatory cytokines and chemokines such as monocyte chemoattractant protein-1 (MCP-1), regulated upon activation, normal T cell expressed and presumably secreted (RANTES) and tumor necrosis factor-α (TNF-α). Thus, platelets are considered to have pro-inflammatory activity [12, 13]. A substantial inflammatory response is linked to venous thrombosis. Acute to chronic changes brought on by this inflammation cause venous wall damage, thrombus organization, and growth. An increase in neutrophils correlates with a rise TNF-α inside the blood vessel wall. Moreover, a constant increase of cytokines in vein walls, such as MCP-1, was observed. These cytokines are important mediators of inflammation, and can trigger the release of matrix metalloproteinases (MMPs), which could cause the deterioration of endothelial cell integrity and apoptosis. In addition to contributing to thrombus development, the inflammatory response is associated with increased platelet activation and aggregation. Other cytokines, including platelet-derived growth factors, also contribute to this inflammatory response. In the second stage of hemostasis, coagulation factors stimulated in cascade, intrinsically and extrinsically, lead to thrombus formation. First, a platelet plug is formed, further supported by a fibrin clot. The clotting cascade involves the activation and interaction of a series of specific blood proteins known as coagulation factors which activate each other in a series of steps. The cascade begins with Factor XII (Hageman factor) activation by contact with collagen or other tissue surfaces. Then, this leads to the activation of Factor XI, which in turn activates Factor IX, and so on, until finally, Factor X is activated, leading to thrombin formation. Thrombin then converts fibrinogen to fibrin, which forms an insoluble mesh-like structure that traps red blood cells, platelets, and other debris to form a clot. Elevated thrombin and fibrinogen levels are connected with thrombosis, which is crucial for platelet aggregation and clot formation. Thrombin also contributes to the production of inflammatory mediators, including TNF-α [14].

Several therapies are used to prevent thrombotic diseases, such as myocardial infarction, deep-vein thrombosis, stroke (embolic or in situ), and pulmonary embolism. Based on the mode of action, there are anti-thrombotic agents (low-molecular-weight heparin) and indirect thrombolytic (plasminogen activator). Despite their efficacy, these agents’ limits are represented by the risk of bleeding [15]. As reported in the scientific literature, reducing inflammation may reduce the risk of cardiovascular disease [9]. However, this is an unexplored area in terms of both prevention and treatment. No previous study has evaluated *Lavandula angustifolia* oil as being used in experimental induced thrombosis.

We aimed to evaluate the efficacy in terms of antioxidant, anti-inflammatory and antithrombotic effects of *Lavandula angustifolia* oil by comparing three different routes of administration at similar doses in experimentally induced venous thrombosis.

## Materials and methods

### Study design

Fifty Wistar Bratislava albino male rats 16 weeks old with tails longer than 13 cm from the Animal Department of Faculty of Medicine, Iuliu Haţieganu University of Medicine and Pharmacy Cluj-Napoca weighing between 300 and 400 g were kept in inscribed (by group name) polypropylene cages at constant temperature (24 ± 2 °C), humidity (60 ± 5%), and light-dark regime, at the Pathophysiology Department. The animals had free access to standard water ad libitum and pellets as diet (Cantacuzino Institute, Bucharest, Romania). The randomization of the animals was made by laboratory personnel who was blinded to the study groups.

**Fig. 1:**
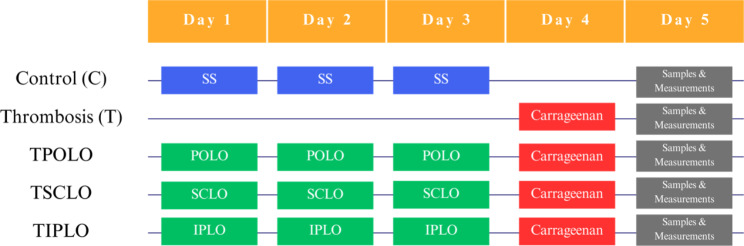
Distribution of group and treatments. Control group (C) was injected with saline solution (SS) 0.9% 1 ml i.p., and DMSO (Dimethylsulfoxide) as vehicle; Thrombosis (T) was induced in the fourth day of the experiment with k-carrageenan 4 mg/kg b.w. (b.w. = body weight), 1 ml i.v. (intravenous) in T, TPOLO, TSCLO and TIPLO group; TPOLO group was pretreated with Lavender oil per os-POLO (150 mg/kg b.w.) and vehicle; TSCLO group received pretreated with Lavender oil subcutaneously-SCLO (150 mg/kg b.w.) and vehicle; TIPLO group received pretreated with Lavender oil intraperitoneal-IPLO (150 mg/kg b.w.) and vehicle

The rats were randomly divided into five groups of ten rats/group (Fig. 1). The animals were pretreated for three days before the induction of thrombosis. Kappa carrageenan is part of a heterogeneous mixture of sulfated polygalactans with pro-inflammatory properties that have long been used in previous studies to create experimental models of inflammation on laboratory rats, but also other pathologies such as peritonitis, local edema, pleurisy, and thrombosis [16]. Previous studies of the carrageenan-induced thrombosis model in the rat tail demonstrated that following histological analysis, carrageenan-induced tail gangrene is actually thrombosis [17]. In the fourth day of the experiment, we dissolved 4 mg/kg b.w. of κ-carrageenan in saline and injected it into the tail vein after ligation 13 cm from the tip of the rat tail. After 10 min, the ligation was removed, thus following the updated method of thrombosis induction according to Hagimori et al. [18].

Carrageenan-induced thrombosis was performed under diethylether anesthesia. Dimethylsulfoxide (DMSO) 5% used as a vehicle for Lavender oil.

Lavender oil, DMSO, k-carrageenan, and ELISA kits were purchased from Sigma-Aldrich (St. Louis, USA).

The animal experiments were conducted in accordance with the protocols approved by the Ethics Committee of the “Iuliu Haţieganu” University of Medicine and Pharmacy Cluj-Napoca (AVZ82/29.03.2022) and the study had approval from the National Sanitary Veterinary and Food Safety Authority, Cluj Branch (Approval no. 135/24.05.2022).

### Measurements

Under light anesthesia with xylazine (2 mg/kg b.w., i.p.) and ketamine (20 mg/kg b.w., i.p.), the blood samples were collected in heparinized tubes from the retro-orbital plexuses of each rat, at 24 h after thrombosis induction. Plasma samples were obtained by centrifugation at 4 C for 20 min at 1620 (×g). The obtained plasma was further transferred in Eppendorf tubes and kept at − 80 °C until further analysis. The animals were sacrificed by an overdose of anesthetics at the end of the experiment.

The evaluation of oxidative stress activity was measured following levels of malondialdehyde (MDA), NO synthesis (NOx), and total oxidative capacity (TOS). The antioxidant capacity of the blood was measured following levels of thiol and total oxidative status (TAC). All the spectroscopic measurements were performed using TECAN SUNRISE spectrophotometer. The plasma levels of the inflammatory cytokines (TNF-α, RANTES, and MCP-1) were measured using the enzyme-linked immunosorbent assay (ELISA) technique. Bleeding and coagulation time were measured using a chronometer by the same researcher. In order to evaluate renal function and hepatic function was measured creatinine, urea, alanine aminotransferase (ALT), and aspartate aminotransferase (AST).

Measurement of the length of the infarcted regions was made at the tip of the tails using a graduated ruler at 24 h after injection of κ-carrageenan.

Skin fragments were collected from the rat tail (fragment of 3 cm at a distance of 5 cm of the tip tail) and fixed for four days in 10% formalin. Afterward, these were included in paraffin, and 5 μm thick sections were made. The sections were stained by the Goldner’s trichrome method and, after, were examined at the optical microscope.

### Statistical methods

Plasma levels of the evaluated markers were reported as median [Q1 to Q3] (Q1 is the 25^th^ percentile and Q3 is the 75^th^ percentile) and {min to max} (min is the smallest value and max is the highest value) to appropriately show the dispersion of data considering the small number of rats per group. The overall significance of measurements was assessed with the Kruskal-Wallis test and whenever statical significance was observed, a posthoc analysis was done to identify differences between the two groups. The measured marker variation was graphically represented using box and whisker plots. Microsoft Office Excel 365 (Microsoft Excel) was used to calculate descriptive statistics and to plot the experimental data. The exploratory inferential analysis was conducted with Statistica program (v. 13.5, StatSoft, St Tulsa, OK, USA); all tests were two-tailed and a *p*-value smaller than 0.05 was considered statistically significant.

## Results

Analysis was conducted on all rats in each group.

### Effect of lavender oil on liver and renal functions

Thrombosis was associated with elevated levels of creatinine, urea, ALT and AST, but significant level was reached only for creatinine (C compared with T, Table 1). Pretreatment with Lavender oil prevented the increase of creatinine, urea and ALT and was induced decreased values of AST in a manner dependent on the form of administration (Table 1).

**Table 1:** Plasma levels of creatinine, urea, ALT, and AST by groups

Group	Creatinine, mg/dL	Urea, mg/dL	ALT, UI	AST, UI
C	0.83 [0.79 to 0.9] {0.72 to 1.01}	49.25 [47.54 to 50.81] {45.03 to 56.11}	47.69 [43.12 to 50.01] {39.31 to 50.91}	39.78 [38.11 to 43.59] {30.42 to 46.9}
T	0.94 [0.93 to 1.05]^a1^ {0.9 to 1.19}	52.24 [49.24 to 55] {46.31 to 60.46}	54.98 [53.95 to 59.75] {52.34 to 67.52}	48.77 [45.95 to 52.15] {43.42 to 57.52}
TPOLO	0.88 [0.85 to 0.9] {0.82 to 1}	47.21 [44.54 to 47.99] {43.74 to 48.24}	41.59 [38.52 to 43]^c3^ {34.84 to 45.75}	27.7 [24.79 to 33.73]^d2^ {22.33 to 46.9}
TSCLO	0.86 [0.85 to 0.92] {0.81 to 0.94}	44.06 [43.26 to 45.51]^b1,b3^ {43.1 to 46.96}	34.46 [32.36 to 38.26]^c1,c4^ {31.43 to 40.54}	25.51 [24.77 to 28.92]^d1,d3^ {22.5 to 33.85}
TIPLO	0.83 [0.8 to 0.86]^a2^ {0.79 to 0.89}	44.06 [42.99 to 45.03]^b2, b4^ {42.45 to 47.19}	30.08 [26.75 to 33.79]^c2,c5^ {24.38 to 36.43}	31.06 [27.2 to 37.08]^d4^ {22.18 to 38.1}
Stat. (p-value)	21.7 (0.0002)	31.4 (< 0.0001)	41.7 (< 0.0001)	33.1 (< 0.0001)

Data are summarize as median [Q1 to Q3] and {min to max}, where Q1 is the 25^th^ percentile, Q3 is the 75^th^ percentile, min is the minimum and max is the maximum value

C = Control group; T = Thrombosis group; TPOLO = Lavender oil Per Os group; TSCLO = SubCutaneously Lavender Oil group; TIPLO = IntraPeritoneal Lavender Oil group; ALT = Alanine aminotransferase ; AST = Aspartate aminotransferase ; Stat. = the statistics of the Kruskal-Wallis test

Post-hoc analysis: ^a1^ 0.0016 C vs. T; ^a2^ 0.0003 T vs. TIPLO; ^b1^ 0.0085 C vs. TSCLO; ^b2^ 0.0029 C vs. TIPLO; ^b3^ 0.0003 T vs. TSCLO; ^b4^ <0.0001 T vs. TIPLO; ^c1^ 0.050 C vs. TSCLO; ^c2^ 0.0008 C vs. TIPLO; ^c3^ 0.0227 T vs. TPOLO; ^c4^ <0.0001 T vs. TSCLO; ^c5^ <0.0001 T vs. TIPLO; ^d1^ 0.0078 C vs. TSCLO; ^d2^ 0.0003 T vs. TPOLO; ^d3^ <0.0001 T vs. TSCLO; ^d4^ 0.0022 T vs. TIPLO

### Effect of lavender oil on oxidative stress and inflammatory cytokines

**Fig. 2:**
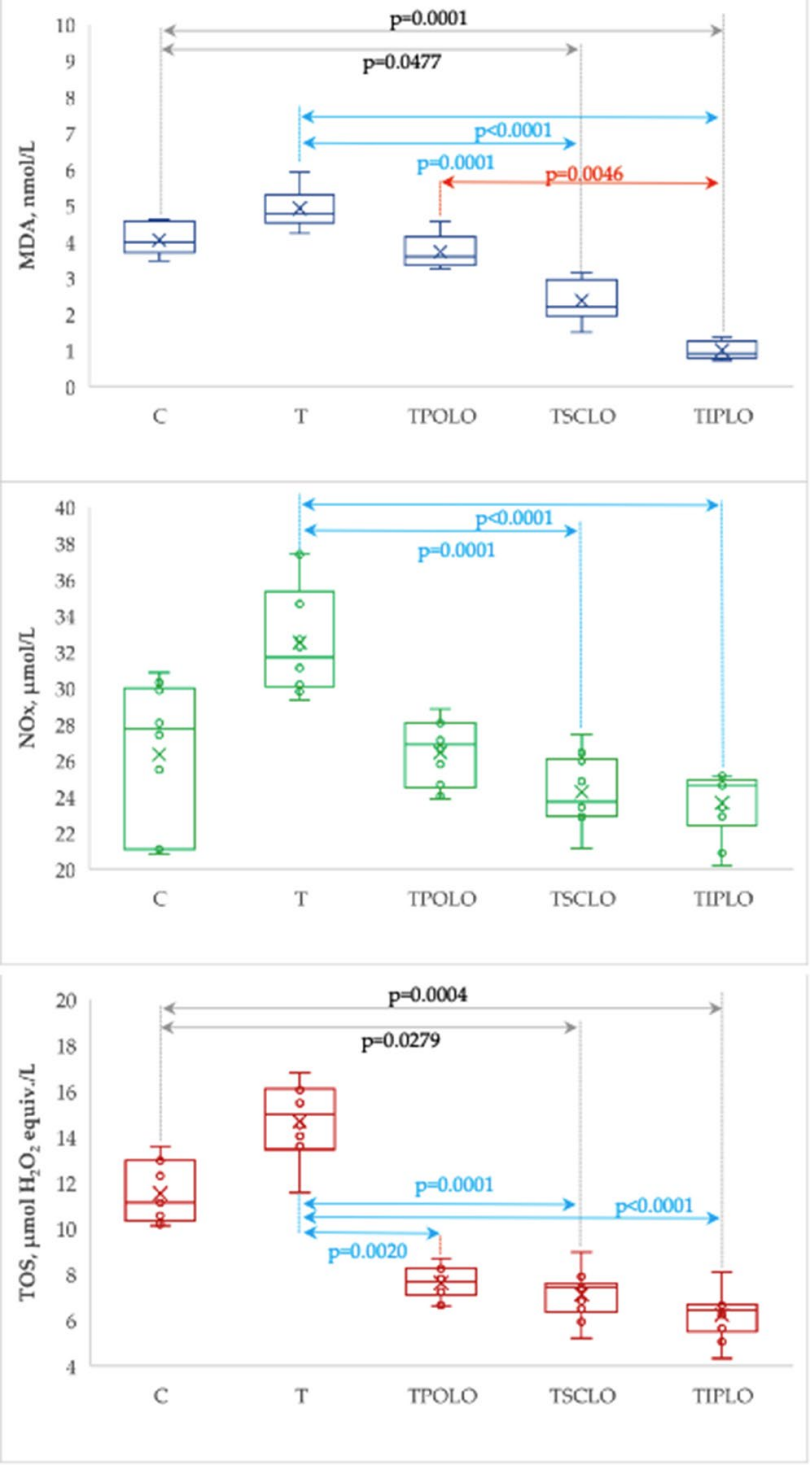
Plasma levels of oxidative stress markers by group. (C = Control group; T = Thrombosis group; TPOLO = Lavender oil Per Os group; TSCLO = SubCu-taneously Lavender Oil group; TIPLO = IntraPeritoneal Lavender Oil group)

The evaluated oxidative stress markers exhibit variability between groups with statistically significant differences compared to the T group (Table 2; Fig. 2).

**Table 2:** Plasma levels of oxidative stress markers by groups

Group	MDA, nmol/L	NOx, µmol/L	TOS, µmol H_2_O_2_ equiv./L
C	3.99 [3.77 to 4.47] {3.47 to 4.62}	27.73 [22.19 to 29.5] {20.82 to 30.84}	11.13 [10.43 to 12.79] {10.12 to 13.59}
T	4.77 [4.57 to 5.2] {4.23 to 5.92}	31.69 [30.2 to 34.16] {29.34 to 37.38}	14.98 [13.7 to 15.9] {11.56 to 16.79}
TPOLO	3.59 [3.36 to 4.05] {3.26 to 4.55}	26.86 [24.94 to 27.85] {23.87 to 28.86}	7.66 [7.22 to 8.13] {6.62 to 8.69}
TSCLO	2.2 [2.02 to 2.9] {1.5 to 3.15}	23.71 [23.03 to 25.7] {21.14 to 27.41}	7.44 [6.58 to 7.51] {5.2 to 8.96}
TIPLO	0.91 [0.78 to 1.22] {0.72 to 1.37}	24.64 [23.03 to 24.84] {20.18 to 25.16}	6.43 [5.77 to 6.64] {4.33 to 8.09}
Stat. (p-value)	43.8 (< 0.0001)	27.3 (< 0.0001)	40.3 (< 0.0001)

Data are summarized as median [Q1 to Q3] and {min to max}, where Q1 is the 25^th^ percentile, Q3 is the 75^th^ percentile, min is the minimum and max is the maximum value

C = Control group; T = Thrombosis group; TPOLO = Lavender oil Per Os group; TSCLO = SubCutaneously Lavender Oil group; TIPLO = IntraPeritoneal Lavender Oil group; MDA = Malondialdehyde; NOx = Nitric Oxide Synthesis; TOS = total oxidative capacity;  Stat. = the statistics of the Kruskal-Wallis test

**Fig. 3:**
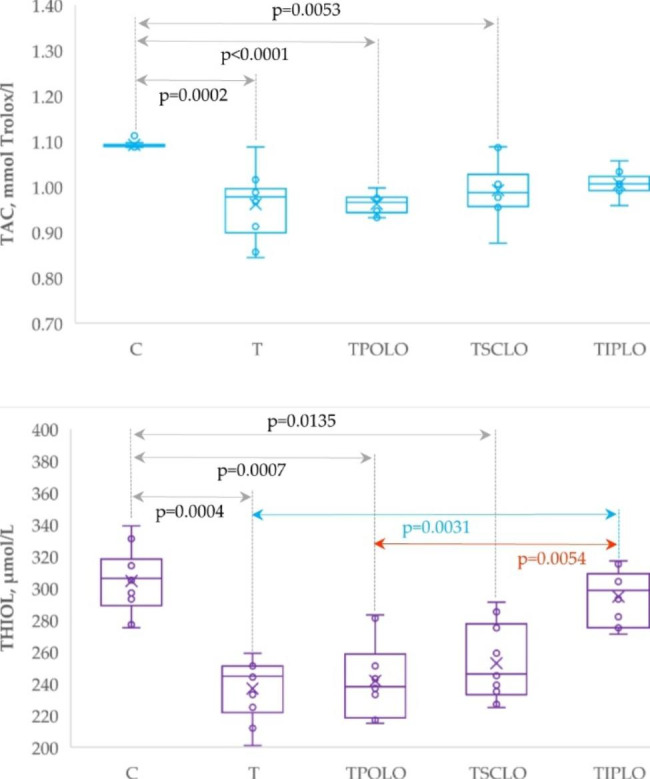
Plasma levels of oxidative stress markers by group. (C = Control group; T = Thrombosis group; TPOLO = Lavender oil Per Os group; TSCLO = SubCutaneously Lavender Oil group; TIPLO = IntraPeritoneal Lavender Oil group)

The closest plasma levels of TAC and thiol by the control group were observed in case of systemic administration of Lavender oil (s.c. and i.p.; Table 3; Fig. 3).

**Fig. 4:**
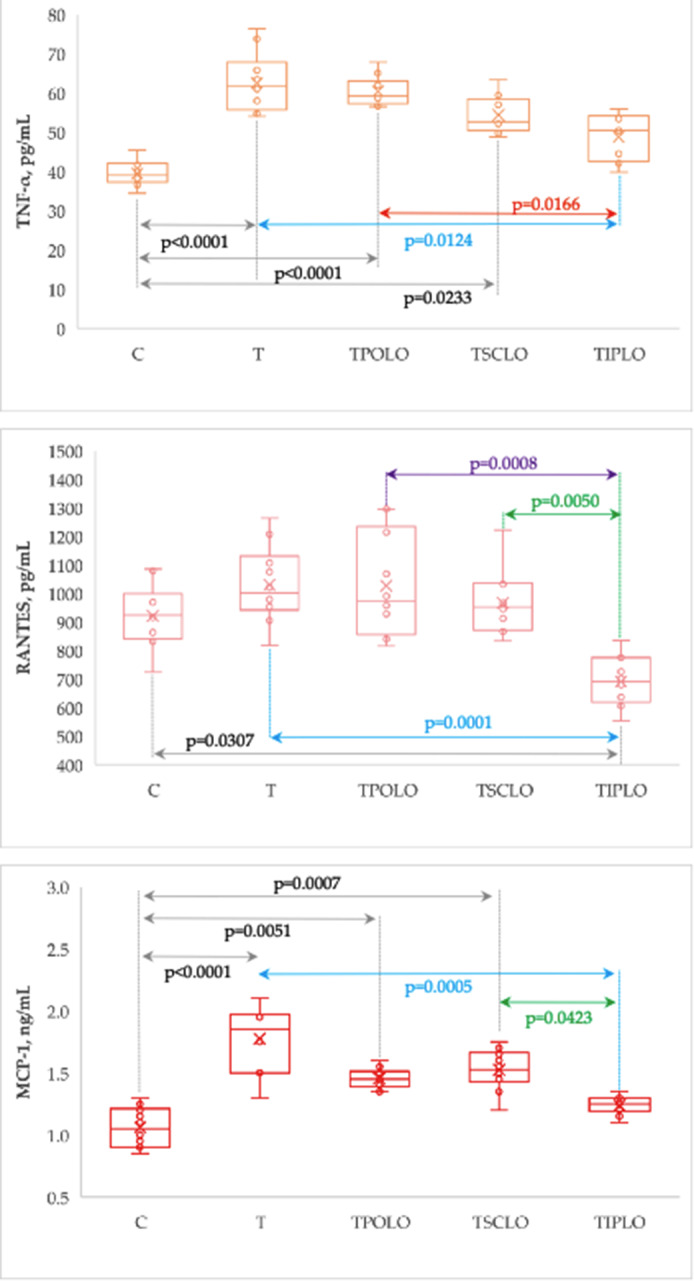
Plasma levels of measured inflammatory cytokines by group. C = Control group; T = Thrombosis group; TPOLO = Lavender Oil Per Os group; TSCLO = SubCutaneously Lavender Oil group; TIPLO = IntraPeritoneal Lavender Oil group)

The plasma levels of TNF-α and MPC-1 significantly increased in T, TPOLO and TSCLO groups as compared with the control group while the group with i.p. pre-treatment exhibit the closest values to the C group (Table 4; Fig. 4).

**Table 3:** Plasma levels of antioxidant capacity and thiol

Group	TAC, mmol Trolox/l	THIOL, µmol/L
C	1.09 [1.09 to 1.09] {1.09 to 1.11}	306 [294 to 312.25] {275 to 339}
T	0.98 [0.93 to 0.99] {0.84 to 1.09}	244.5 [227 to 250] {201 to 259}
TPOLO	0.97 [0.95 to 0.98] {0.93 to 1}	238 [222.5 to 249] {215 to 283}
TSCLO	0.99 [0.96 to 1.01] {0.88 to 1.09}	246 [236 to 271] {225 to 291}
TIPLO	1.01 [0.99 to 1.02] {0.96 to 1.06}	298.5 [276.75 to 307] {271 to 317}
Stat. (p-value)	29.3 (< 0.0001)	30.8 (< 0.0001)

Data are summarized as median [Q1 to Q3] and {min to max}, where Q1 is the 25^th^ percentile, Q3 is the 75^th^ percentile, min is the minimum and max is the maximum value; C = Control group; T = Thrombosis group; TPOLO = Lavender oil Per Os group; TSCLO = SubCutaneously Lavender Oil group; TIPLO = IntraPeritoneal Lavender Oil group;  Stat. = the statistics of the Kruskal-Wallis test

**Table 4:** Plasma levels of inflammatory cytokines

Group	TNF-α, pg/mL	RANTES, pg/mL	MCP-1, ng/mL
C	39.07 [37.57 to 41.3] {34.38 to 45.42}	923.45 [848.06 to 970.8] {724.55 to 1085.3}	1.05 [0.91 to 1.19] {0.85 to 1.3}
T	61.68 [56.53 to 65.2] {53.99 to 76.35}	1000.2 [958.35 to 1098.6] {817.2 to 1263.8}	1.85 [1.56 to 1.97] {1.3 to 2.1}
TPOLO	59.18 [57.6 to 62.26] {56.5 to 67.77}	974.05 [876.96 to 1177.65] {815.5 to 1294.9}	1.45 [1.4 to 1.5] {1.35 to 1.6}
TSCLO	52.67 [50.74 to 57.81] {48.77 to 63.35}	952.2 [881.88 to 1015.81] {832.75 to 1223}	1.53 [1.46 to 1.64] {1.2 to 1.75}
TIPLO	50.46 [43.15 to 53.78] {39.77 to 55.91}	690.18 [627.01 to 762.75] {552.95 to 835.85}	1.25 [1.2 to 1.3] {1.1 to 1.35}
Stat. (p-value)	35.7 (< 0.0001)	23.8 (0.0001)	36.6 (< 0.0001)

Data are summarized as median [Q1 to Q3] and {min to max}, where Q1 is the 25^th^ percentile, Q3 is the 75^th^ percentile, min is the minimum and max is the maximum value. C = Control group; T = Thrombosis group; TPOLO = Lavender oil Per Os group; TSCLO = SubCu-taneously Lavender Oil group; TIPLO = IntraPeritoneal Lavender Oil group;  Stat. = the statistics of the Kruskal-Wallis test

### Effect of lavender oil on thrombus length, bleeding and clothing time

**Fig. 5:**
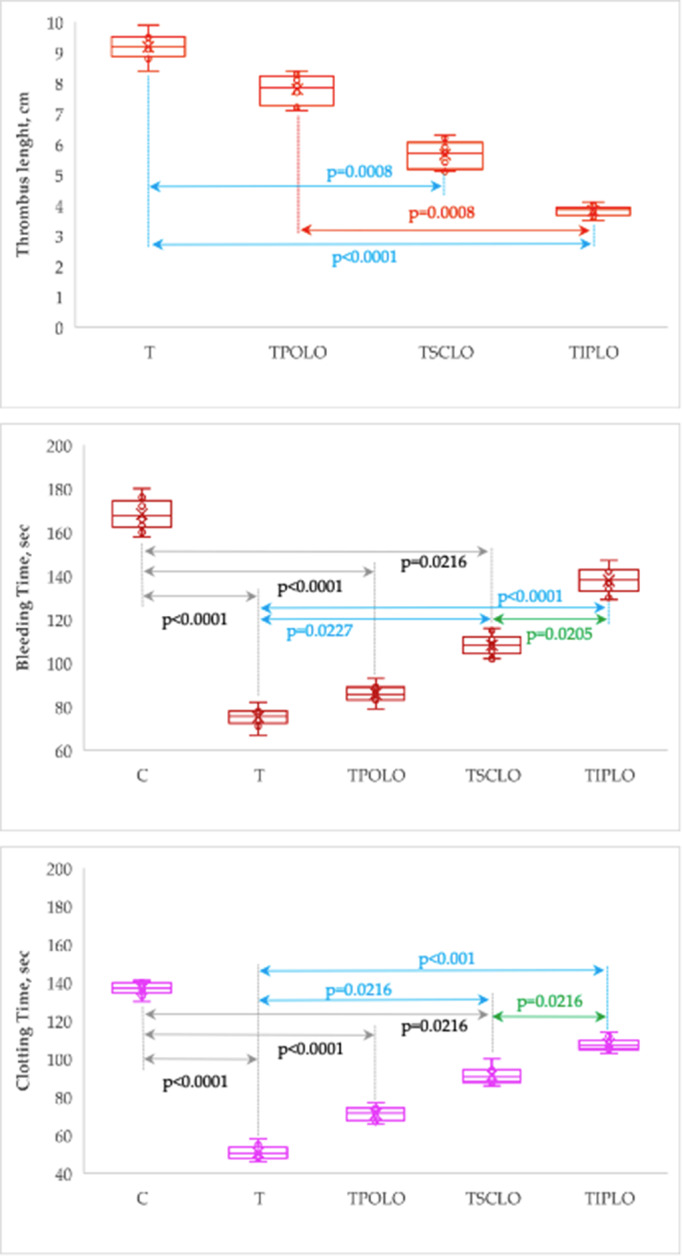
Thrombus length, bleeding and clotting time by group. (C = Control group; T = Thrombosis group; TPOLO = Lavender oil Per Os group; TSCLO = SubCutaneously Lavender Oil group; TIPLO = IntraPeritoneal Lavender Oil group)

Pretreatment with lavender oil influences the thrombus length, bleeding, and clotting time in an administration type manner, with the best results obtained by i.p. administration (Table 5; Fig. 5).

**Table 5:** Variation of thrombus length, bleeding, and clotting time by group

Group	Length, cm	Bleeding Time, sec	Clotting Time, s
C		167.5 [163.75 to 173.5] {158 to 180}	137 [135 to 139.75] {130 to 141}
T	9.2 [8.9 to 9.48] {8.4 to 9.9}	75.5 [73.25 to 78] {67 to 82}	50.5 [48 to 52.75] {46 to 58}
TPOLO	7.85 [7.4 to 8.18] {7.1 to 8.4}	85.5 [83.25 to 88.75] {79 to 93}	71.5 [68.25 to 73.5] {66 to 77}
TSCLO	5.7 [5.25 to 5.98] {5.1 to 6.3}	108 [105.25 to 110.5] {102 to 116}	90.5 [88.5 to 93.25] {86 to 100}
TIPLO	3.85 [3.73 to 3.9] {3.5 to 4.1}	138 [134.25 to 142.75] {129 to 147}	107 [105.25 to 109] {103 to 114}
Kruskal-Wallis stat. (p-value)	36.5 (< 0.0001)	47.0 (< 0.0001)	47.1 (< 0.0001)

Data are summarize as median [Q1 to Q3] and {min to max}, where Q1 is the 25^th^ percentile, Q3 is the 75^th^ percentile, min is the minimum and max is the maximum value. C = Control group; T = Thrombosis group; TPOLO = Lavender oil Per Os group; TSCLO = SubCutaneously Lavender Oil group; TIPLO = IntraPeritoneal Lavender Oil group

### Efficacy of Lavander Oil on histopathological changes

**Fig. 6:**
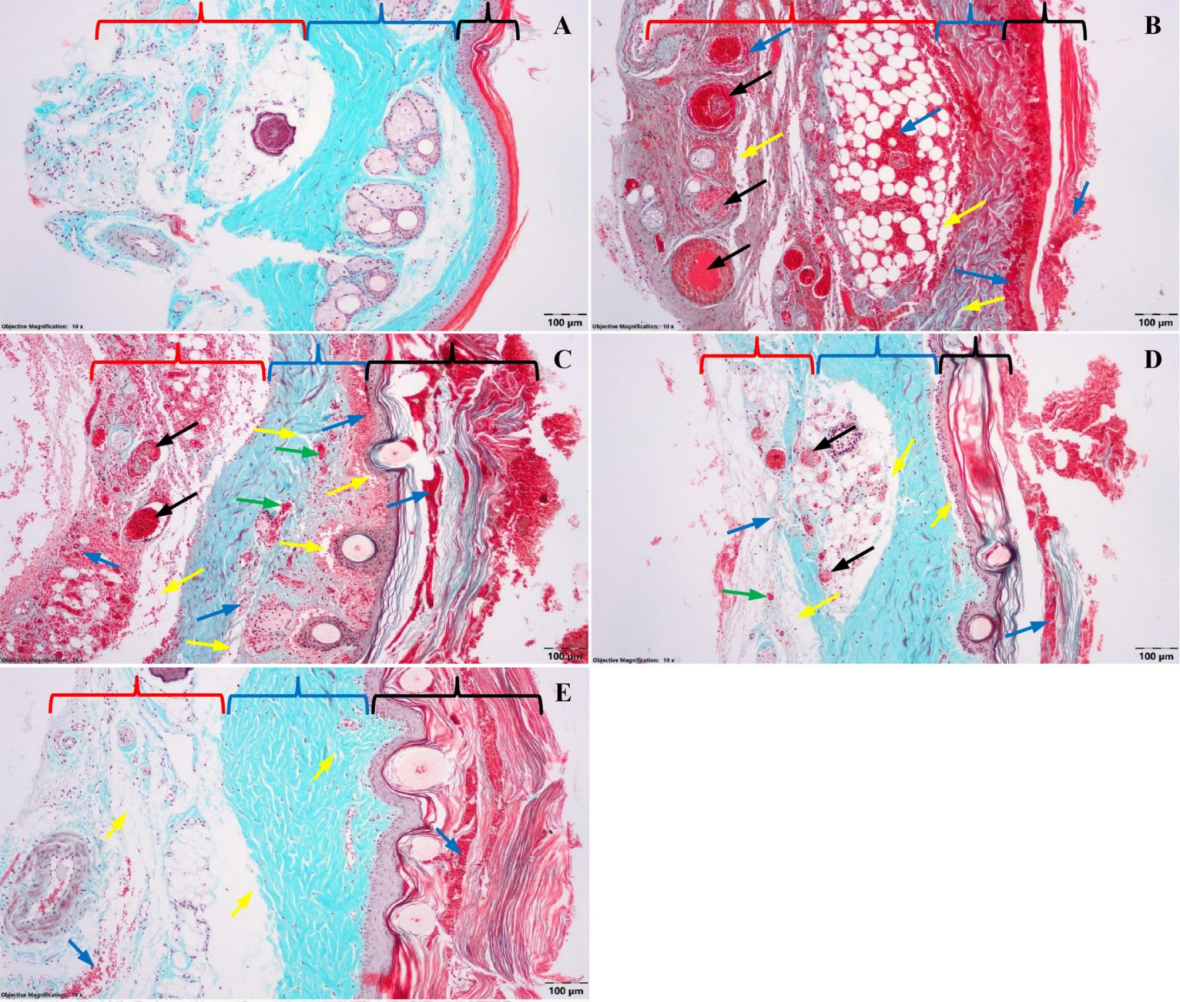
Histopathological examination of the tails. **A**) – Control (**C**) group; **B**) – Thrombosis (T) group; (C) – Lavender oil per os (TLOPO) group; (D) – Subcutaneously Lavender Oil (TSCLO) group; (E) – Intraperitoneal Lavender Oil (TIPLO) group. Goldner’s trichrome stain; red brace – hypodermis; blue brace – dermis; black brace – epidermis; black arrow - thrombosis; blue arrow - haemorrhage; yellow arrow - oedema; green arrow - congestion

No microscopic changes are found in the epidermis, dermis, and hypodermis in the control group (C, Fig. 6A).

In the thrombosis (T) group, several pathological aspects are present (Fig. 6B) in all layers of the skin. In the hypodermis, the lumen of numerous veins and venules appears completely or partially obliterated by thrombi that have formed. Following the obliteration of blood circulation in the venous system, massive capillary stasis with excessive dilatation is observed in the lax connective tissue from the hypodermis. There are also numerous portions of transferred plasma in different stages of coagulation and numerous red blood cells. The transferred red blood cells also invade the adipose cells in the hypodermis. At the level of the deep dermis, can also be observed veins in which intravascular coagulation and red blood cells are undergoing the lysis phenomenon. In dense connective tissue, the spaces between the collagen fibers are more prominent due to the increase of the tissue fluid. In the superficial dermis, massive dilation of small blood vessels and numerous transferred red blood cells can be observed. Large amounts of liquid and numerous red blood cells appear accumulated under the basal membrane of the epidermis, which is separated in some places from the superficial dermis. In the epidermis, among the keratinocytes, numerous red blood cells, especially in the superficial layer, are noticed, which leads to the separation of the keratin layers. Due to the edema and subepidermal hemorrhages, numerous keratinocytes from the spinous layer and some from the basal layer of the epidermis show specific aspects of the first stages of cell death among the nucleus of the cells.

The microscopic aspects (Fig. 6C) are similar in the TPOLO group to those in the thrombosis (T) group with some minor differences. Specifically, the number of veins with a completely obliterated lumen in the hypodermis is significantly lower, but numerous hemorrhages can also be observed at this level. At the junction between the hypodermis and the deep dermis, there are large areas in which large amounts of liquid have torn apart the two layers. In the deep dermis, there are rare red blood cells in the fundamental substratum. The amount of liquid in the fundamental substance of the deep dermis is significantly lower than in the thrombosis group because the distance between the collagen fibers is thinner. At the level of the superficial dermis, some lesions are much more significant than those in the deep dermis. Thus, large areas are covered by interfibrillar edema and numerous diffuse hemorrhages. There are also portions of blood in various stages of coagulation and hemolysis under the epidermis’s basal membrane, which is also detached from the dermis at this level. Compared to thrombosis group, the number of red blood cells reaching the conjunctive tissue from the superficial dermis is lower. However, on the other hand, at the epidermis level, especially between the layers of keratinized cells, there is a much higher number of red blood cells. In the deep layers of the epidermis (basal and spinous), the number of cells showing nuclear-specific aspects to the early stages of apoptosis is much smaller.

In the hypodermis of the TSCLO group small veins with obliterated lumen could be observed (Fig. 6D). Furthermore, the hemorrhage is much less in the TSCLO group compared to T and TPOLO groups. Instead, there is a pronounced edema at the junction between the dermis and the hypodermis. In the deep dermis, there are no changes, but in the superficial dermis, there is interfibrillar edema along with small hemorrhages. At the junction between the dermis and the epidermis, there are relatively large areas where the liquid has broken the attachment of the basal membrane by the dermis, and in the created spaces, there are rare red blood cells from place to place. In the epidermis, in the areas where the basal membrane is detached from the dermis, numerous cells from the basal layer show pyknotic nuclei or nuclei with thickened nuclear membranes. Also, among the layers of keratinized cells from the superficial layer of the epidermis, there are red blood cells in different stages of lysis, and superficially between the rows of keratinocytes, there are spaces created by the large amounts of liquid pushed from the dermis.

A pronounced edema and rare extravasated red blood cells are observed in the hypodermis of the TIPLO group (Fig. 6E). The liquid tends to accumulate at the dermis junction, where relatively large spaces are created. In the dermis, there are no changes. From place to place, there are tiny areas where the basal membrane of the epidermis tends to be torn apart from the dermis. In the epidermis’s deep layers (basal and spinous), no nuclear aspects suggest cellular death. However, on the other hand, in the superficial layer, numerous red blood cells are present among the keratinocytes.

## Discussion

Our study demonstrated that intraperitoneal administration of *Lavandula Angustifolia* oil as a pretreatment has the best effects on thrombosis and its associated inflammation. Lavender oil administration resulted in low dimensions of thrombosed tail length. The group pretreated with lavender oil by intraperitoneal route (TIPLO group) showed the smallest length of the thrombosis size (Table 5). These differences were significant compared to the group of rats with thrombosis (T group), and the group treated orally with lavender oil (TPOLO group). The tail thrombosis lengths of rats treated with lavender oil administered intraperitoneally (TSCLO) were likewise smaller than those of rats treated with LO administered subcutaneously (TSCLO), but the differences do not reach statistical significance. In comparison to the T group, the thrombosed tail region lengths of the TSCLO group were shorter. Animals that received the pretreatment by oral route did not display any significant changes in comparison to those without any pretreatment (T group). (see Table 5; Fig. 5) Intraperitoneal route proved to be the most effective route of administration, resulting in a significant reduction of both bleeding time and clotting time. Although the subcutaneous route achieved a significant reduction in bleeding and clotting time, the intraperitoneal route was significantly superior (Table 5; Fig. 5). The intraperitoneally pretreated rats had bleeding and clotting times close to the values of the control group, without statistical significance from the saline group. On the other hand, the TPOLO group did not show any statistically significant differences from the thrombosis group (T) regarding this assessment (Table 5; Fig. 5). Moreover, the results of our research demonstrate that intraperitoneal pretreatment of 150 mg/kg b.w. of *Lavandula angustifolia* oil increases the bleeding and clotting time close to normal values in an animal experimental model of thrombosis, without increasing the risk of spontaneous bleeding. *Lavandula hybrida* oil’s effects, including linalool and linalyl acetate as its principal components, were evaluated in a previous in vitro study (7) that showed antiplatelet and antithrombotic activities effects of the *Lavandula* oil. Major components of the *Lavandula hybrida* were able to inhibit the aggregation obtained with arachidonic acid, U46619, and collagen [7]. In the same research, it was counted the amount of blood loss. They were using the tail transection bleeding Dejana’s method. Mice treated with 100 mg/body weight of lavender oil showed no significant difference from control losing blood [7].

Pathological investigations indicate that k-carrageenan can induce local blood vessel inflammation and endothelial cell damage by releasing inflammatory factors, which may result in thrombus development [19]. It is considered that inflammation, particularly in blood vessels, is a factor in developing thrombosis. Nonetheless, blood clots may also lead to inflammation. Hence, bidirectional interaction between inflammation and thrombosis plays a significant role in k-carrageenan-induced thrombosis. Carrageenan-induced thrombosis may entail mechanisms such as the activation of phospholipase A2, the production of inflammatory mediators, cytokines, and the coagulation cascade activation. The release of thrombotic factors, such as fibrinogen, factor V, and thrombin, can activate inflammatory pathways, leading to an increased inflammatory response. In addition, k-carrageenan can enhance the production of adhesion molecules, which might assist in the deposition of platelets and leukocytes, which could contribute to thrombus development [20]. Previous studies have demonstrated the importance of oxidative-antioxidative balance in thrombosis and the correlation between oxidative stress imbalance and thrombosis [21, 22]. It has also been shown that k-carrageenan is a pro-inflammatory compound widely used in experimental animal models [23].

In our study, oxidative stress parameters were improved, especially in rats from the TIPLO group (Table 2; Fig. 2). The circulating levels of MDA and NOx were significantly decreased for the groups of rats treated intraperitoneally and subcutaneously with lavender oil. Rats treated intraperitoneally had significantly lower MDA levels than those treated *per os*. The only oxidative stress parameter that was measured and showed significant changes in all routes of administration was TOS (Table 2; Fig. 2). Regarding the antioxidant markers, TAC and Thiol, the TIPLO and TSCLO groups had increased values compared to the thrombosis group (Table 3; Fig. 3). However, the intraperitoneally pretreated rats had the closest values to the control group without statistically significant differences. Malondialdehyde (MD) is one of the most widely used biomarkers of oxidative stress. In vivo studies have used MDA levels to measure the degree of oxidative stress in a given system [24]. According to findings from earlier studies, the administration of lavender oil to rats reduces the amount of MDA found in both the rats’ serum and brain tissue [25]. Also, an in vitro study showed that lavender oil could reduce nitric oxide synthesis (NOx) levels [26]. These findings may be attributed to lavender’s antioxidant and anti-inflammatory properties, which have been previously reported. Lavender extract is thought to scavenge free radicals, increase the activity of antioxidant enzymes such as superoxide dismutase (SOD) and glutathione peroxidase (GSH-Px), and reduce the production of pro-inflammatory cytokines [27]. Reducing oxidative stress and inflammation may contribute to the protective effects of lavender oil on thrombosis.

Cytokines that promote inflammation are known as proinflammatory cytokines. Many researchers believe they represent a crucial connection between inflammation and the hypercoagulable, prothrombotic state that may be seen in specific clinical circumstances [28]. Together, these cytokines may enhance an inflammatory response, increasing the synthesis of additional proinflammatory molecules, including chemokines, proteases, and reactive oxygen species. Chemokines may develop in tissue damage and the development of blood clots due to a cascade of physiological changes. Therefore, understanding the role of these cytokines and their role in inflammation can be essential for developing therapies for various conditions.

Our study showed significant changes in TNF alpha, RANTES, and MCP-1 in rats pretreated intraperitoneally with lavender oil compared to T group rats (Table 4; Fig. 4). Moreover, the TIPLO group had significantly lower values of all three inflammatory parameters evaluated than the orally treated TPOLO group. Also, compared to the TSCLO group, rats pretreated intraperitoneally with lavender oil showed significantly lower values for RANTES and MCP-1. Tumor necrosis factor-α is a multifunctional cytokine important in immune regulation, inflammation, thrombosis, and tumor metastasis [29]. It is produced by various cells, including T cells, B cells, macrophages, and fibroblasts. It is involved in regulating a wide variety of cellular processes, including cell growth, differentiation, apoptosis, and immune responses. The TNF-α cytokine can be either pro-inflammatory or anti-inflammatory. In its proinflammatory form, it can induce inflammation, activate immune cells, and produce other proinflammatory cytokines. In its anti-inflammatory form, it can reduce inflammation and regulate the production of other cytokines. This cytokine has been implicated in the pathogenesis of several diseases, including rheumatoid arthritis, psoriasis, inflammatory bowel disease, and cancer [30]. The capacity of TNF-α to upregulate tissue factor, downregulate thrombomodulin, decrease the density of endothelial protein C receptors, and block fibrinolysis on endothelial cells indicates a function for cytokines in mediating inflammation-induced coagulation/thrombosis [28]. Monocyte chemoattractant protein-1 is an essential chemokine that plays a vital function in the inflammatory process by enhancing the production of other inflammatory factors/cells. This primary method of migration and infiltration of inflammatory cells at the site of inflammation contributes to the progression of several illnesses. The chemokine MCP-1, which is generated by various cell types, is known to play a role in the attraction of macrophages, monocytes, and T cells. Many pathogenic disorders, including cardiovascular diseases, brain pathologies, bone and joint abnormalities, respiratory infections, cancer, and endothelial dysfunction, are associated with monocyte chemoattractant protein-1 implication. This chemokine is essential for migrating monocytes to the subendothelium and attracting leukocytes to the site of injury, hence enhancing thromboembolic and atherogenic potential [31]. Studies have shown that MCP-1 can activate endothelial dysfunction, an essential factor in the development of coagulopathy [12, 32]. Also, RANTES is a chemokine (C-C motif) ligand 5 that is stored in the α-granules of activated platelets. It is a potent chemoattractant for various cells, including monocytes and other immune cells. When inflammatory cells are recruited and activated, they can produce more inflammatory mediators like cytokines and reactive oxygen species. This process is facilitated by RANTES. Von Willebrand Factor (VWF) is also stored in α-granules and plays a role in platelet adhesion, aggregation, and thrombus formation. In addition to its hemostatic function, VWF has been shown to be involved in vascular inflammation. It has been demonstrated that VWF can bind to receptors on the surface of endothelial cells, inducing the production of proinflammatory molecules, such as tumor necrosis factor-alpha. Von Willebrand Factor can also interact with certain leukocytes, including monocytes and T cells, and activate them, releasing further inflammatory mediators [33].

Regarding hepatotoxicity and renal toxicity, the administration of lavender oil showed no adverse effects. Moreover, subcutaneous administration showed significantly lower values of ALT, AST, and urea compared to the group of rats with thrombosis. However, the only route of administration that decreased all measured parameters, including creatinine, was intraperitoneal administration. Our results are in agreement with the results reported in the scientific literature, namely the lack of hepatic and renal adverse effects following lavender oil administration [34, 35].

Histopathological examination showed important differences between groups pretreated with *Lavandula Angustifolia* oil. No significant differences were observed between the thrombosis group and TPOLO. However, the subcutaneous pretreated group, and especially the intraperitoneal pretreated group, displayed rare extravasated red blood cells, and no nuclear aspects suggest cellular death (Fig. 6).

Our results suggest that pretreatment with lavender oil could be a potential solution to decrease oxidative stress and inflammation in patients at risk of thrombosis without risk of bleeding.

## Conclusions

The results showed that lavender oil effectively reduced thrombus formation and tissue damage and reduced the levels of inflammatory cytokines and oxidative stress in serum. Intraperitoneal administration of Lavandula angustifolia oil as a pretreatment has the best efficacy on thrombosis and its associated inflammation. Our study provides evidence that lavender oil has potential protective effects against thrombosis, likely due to its anti-inflammatory and antioxidant effects. Further studies are needed to confirm these findings and elucidate the mechanisms by which lavender extract exerts its protective effects.

## Data Availability

The raw data analyzed in this study are part of a Ph.D. study and can be obtained upon reasonable request addressed to Valeriu Mihai But (but.valeriumihai@elearn.umfcluj.ro).

## Abbreviations

ALT: Alanine aminotransferase
AST: Aspartate aminotransferase
BW: Body weight
C: Control group
DMSO: Dimethylsulfoxide
HMGB1: High mobility group protein B1
IP: Intraperitoneal
LO: Lavender oil
MCP-1: Monocyte chemoattractant protein-1
MDA: Malondialdehyde
MMPs: Metalloproteinases
NOx: Oxide nitric synthesis
RANTES: Regulated upon activation, normal T cell expressed and presumably secreted
SS: Saline solution
T: Thrombosis group
TAC: Total oxidative status
TIPLO: IntraPeritoneal Lavender Oil group
TLOPO: Lavender oil Per Os group
TNF-α: Tumor necrosis factor-α
TOS: Total oxidative capacity
TSCLO: SubCutaneously Lavender Oil group
VWF: Von Willebrand Factor
